# Professional Confidence—Let It Be Respected in Courts

**Published:** 1882-05

**Authors:** 


### PROFESSIONAL CONFIDENCE -LET IT BE RESPECTED IN COURTS.

---

To argue the sacredness with which the statements, or secrets it may be, made by a patient to his medical adviser should be regarded, would be an insult to the profession. Certainly we can conceive of no confidence which should be more inviolable. Not the physician alone, but the patient, the whole public we may say, appreciate not alone the right, but the absolute necessity for such inviolability. On such appreciation very often depend the health and life of the patient. On it may hang the happiness of individuals and families. Even though there may be confessions of immorality, it is the physician's part to heal—not to administer justice. He, as the representative of a profession almost divine in its sentiment and principle, has received these secrets. To use, or to permit others to use such information for any purpose other than the object for which it was afforded, is dishonor. It is theft; it is the most degrading form of theft. If any power compel him to divulge it, that power is a party to such crime. We are not sure if such power is not the sole, or at least the principal criminal. Such exercise of power, in truth, is doubly wrong. It is a wrong done the patient and a wrong done the physician and his profession. We repeat, the judge or the law, that forces such disclosure, commits the lowest kind of sneak thievery.

We believe an attorney-at law is not required to testify to facts received from a client in his professional capacity. Why is a physician not so protected? A few states, but only a few, have made enactments for this purpose. It is time that all do so.

We have said the law should not compel a physician to so humiliate himself. But can he in reality be so compelled? As a horse may be led to water, but cannot be made to drink, so may a physician be placed on the stand, but only himself can use his tongue. Shall he allow himself to be driven by fear of fine or imprisonment, or by its actual enforcement? We believe he should not. Physicians have gone to prison rather than commit the wrong they were thus asked to do. If we remember correctly, such a course, in one instance, resulted in some healthy legislation on the subject. It might be well for the Medical Association, shortly to assemble in this city, to take some steps toward bringing this matter before the Georgia legislature.

